# Histamine Regulates the Inflammatory Profile of SOD1-G93A Microglia and the Histaminergic System Is Dysregulated in Amyotrophic Lateral Sclerosis

**DOI:** 10.3389/fimmu.2017.01689

**Published:** 2017-11-30

**Authors:** Savina Apolloni, Paola Fabbrizio, Susanna Amadio, Giulia Napoli, Veronica Verdile, Giovanna Morello, Rosario Iemmolo, Eleonora Aronica, Sebastiano Cavallaro, Cinzia Volonté

**Affiliations:** ^1^Experimental Neuroscience, Santa Lucia Foundation, Rome, Italy; ^2^National Research Council, Institute of Cell Biology and Neurobiology, Rome, Italy; ^3^National Research Council, Institute of Neurological Sciences, Catania, Italy; ^4^Department of (Neuro) Pathology, Academic Medical Center, Amsterdam, Netherlands

**Keywords:** amyotrophic lateral sclerosis, histamine, microglia, neuroinflammation, SOD1-G93A

## Abstract

Amyotrophic lateral sclerosis (ALS) is a late-onset motor neuron disease where activated glia release pro-inflammatory cytokines that trigger a vicious cycle of neurodegeneration in the absence of resolution of inflammation. Given the well-established role of histamine as a neuron-to-glia alarm signal implicated in brain disorders, the aim of this study was to investigate the expression and regulation of the histaminergic pathway in microglial activation in ALS mouse model and in humans. By examining the contribution of the histaminergic system to ALS, we found that particularly *via* H1 and H4 receptors, histamine promoted an anti-inflammatory profile in microglia from SOD1-G93A mice by modulating their activation state. A decrease in NF-κB and NADPH oxidase 2 with an increase in arginase 1 and P2Y12 receptor was induced by histamine only in the ALS inflammatory environment, but not in the healthy microglia, together with an increase in IL-6, IL-10, CD163, and CD206 phenotypic markers in SOD1-G93A cells. Moreover, histaminergic H1, H2, H3, and H4 receptors, and histamine metabolizing enzymes histidine decarboxylase, histamine *N*-methyltransferase, and diamine oxidase were found deregulated in spinal cord, cortex, and hypothalamus of SOD1-G93A mice during disease progression. Finally, by performing a meta-analysis study, we found a modulated expression of histamine-related genes in cortex and spinal cord from sporadic ALS patients. Our findings disclose that histamine acts as anti-inflammatory agent in ALS microglia and suggest a dysregulation of the histaminergic signaling in ALS.

## Introduction

The biogenic amine histamine is synthesized by decarboxylation of l-histidine by histidine decarboxylase (HDC) and catabolized by histamine *N*-methyltransferase (HNMT) and diamine oxidase (DAO, or AOC), enzymes that are distributed in all CNS areas. While histamine-producing neurons are found mainly in the hypothalamus, histamine acts as an ubiquitous transmitter and pleiotropic agent, whose signaling is mediated by G protein-coupled receptors such as H1R, H2R, H3R, and H4R (1). In the CNS, histamine regulates the sleep–wake cycle, nociception, motor circuits, satiety signaling, and neuroimmune functions (2, 3). Since its discovery in 1910, histamine gained increasing attention in health and disease, but its role in CNS dysfunction remains to be elucidated.

As proven by human postmortem and animal model studies, the histaminergic system becomes altered in several brain disorders, and histaminergic abnormalities are hypothesized to contribute to neurodegenerative disorders, such as Parkinson’s and Alzheimer’s diseases (2, 4–6). In addition, H3R antagonists/inverse agonists, potentiating the endogenous release of histamine in the CNS, display beneficial effects in animal models of many neurological disorders, and have been administered in patients in clinical trials for schizophrenia, cerebral ischemia, Alzheimer’s and Parkinson’s diseases (6). However, the involvement of histamine is basically unexplored in amyotrophic lateral sclerosis (ALS), a late-onset neurodegenerative/neuroinflammatory disease characterized by progressive loss of motor neurons in the motor cortex, brain stem, and ventral horns of the spinal cord.

Amyotrophic lateral sclerosis is diagnosed as sporadic disease [sporadic ALS (sALS)] in about 90% of all patients, while in the remaining cases of familial origin at least 100 different gain-of-function mutations are found in the *ALS1* gene coding for the SOD1 enzyme, by itself accounting for about 20% of all familial cases (7). ALS has a marked multifactorial nature where genetic factors contribute to aggravate the pathogenesis and possesses a non-cell-autonomous feature characterized by damage to different cell populations contributing to different phases of the disease. For instance, in the CNS, injury to the motor neurons seems to be linked to disease onset, while glial cells, particularly microglia undergoing mutant SOD1-mediated neuroinflammatory activation, are responsible for disease progression and further motor neuron impairment and death (8, 9).

Because histamine is a widespread neuroimmune modulator known to act *in vivo* and *in vitro* (5, 10–13), the aim of this study was to establish the expression and regulation of the histaminergic pathway in inflammatory mechanisms of microglia in ALS mouse model and in humans.

## Materials and Methods

### Reagents

Histamine and all reagents were from Sigma-Aldrich (Italy), unless otherwise stated. JNJ7777120 was from Selleck Chemicals (USA), PD098059 from Calbiochem (USA), ranitidine hydrochloride from R&D system (USA), and thioperamide maleate from Santa Cruz Biotechnology (USA).

### SOD1-G93A Mice

Adult B6.Cg-Tg(SOD1-G93A)1Gur/J mice expressing high copy number of mutant human SOD1 with a G93A substitution (SOD1-G93A) were originally obtained from Jackson Laboratories (USA) and bred as described (14). Animal procedures were performed according to European Guidelines for use of animals in research (2010/63/EU) and requirements of Italian laws (D.L. 26/2014) and approved by the Animal Welfare Office, Department of Public Health and Veterinary, Nutrition and Food Safety, General Management of Animal Care and Veterinary Drugs of the Italian Ministry of Health (protocol number 319/2015PR). All efforts were made to minimize animal suffering and the number of animals necessary to produce reliable results. Transgenic progeny was genotyped as previously described (15). To allow better reproducibility and avoid gender-dependent differences, only female SOD1-G93A mice tracked for their estrous cycle (*n* = 4/group) were sacrificed at 100 (presymptomatic), 140 (symptomatic), and approximately 165 days of age (end stage) (16).

To test the effects of an acute treatment with histidine on inflammatory markers, female SOD1-G93A mice (~140 days of age) were randomly grouped into histidine (500 mg/kg body weight, i.p. single dose) or vehicle (0.9% NaCl) groups (*n* = 4/group). This dose provided detectable levels of histamine in CNS (17–19) and showed efficacy in models of cerebral ischemia (20). After 3 days, the mice were transcardially perfused with 50 ml of PBS and tissue processed as described for immunofluorescence (IF) or western blotting (WB) analysis. Previous data reported modifications in microgliosis at 3 days after histamine treatment in mice (5).

### Primary Microglia Cultures

Microglia cultures from mouse brain cortex were prepared as previously described (21). Briefly, neonatal SOD1-G93A and wild-type (WT) mice were sacrificed and, after removing the meninges, cortices were digested with 0.01% trypsin and 10 µg/ml DNaseI. After dissociation and passage through 70-µm filters, cells were plated onto 100-mm culture dishes and cultured in DMEM/F-12 media (Gibco, Invitrogen, UK), plus 10% fetal bovine serum, 100 U/ml gentamicin, and 100 µg/ml streptomycin/penicillin. After approximately 15 days, a mild trypsinization was done to remove non-microglial cells. The resultant adherent microglial cells (~99% pure) were replated onto culture dishes as described in Section “Cell Treatments” and kept in 50% glial cells conditioned medium at 37°C in a 5% CO2 and 95% air atmosphere for 48 h before use.

### Cell Treatments

Cells were plated at a density of 1 × 10^5^ cells/well in 12-well plate (WB), 1 × 10^5^ cells/35 mm dish (IF assay), and 2 × 10^5^ cells/35-mm dish (RT-PCR and migration assay). Cell treatments included the following: histamine (100 µM), H1 receptor antagonist orphenadrine (10 µM), H2 receptor antagonist ranitidine (10 µM), H3 receptor antagonist thioperamide (5 µM), H4 receptor antagonist JNJ7777120 (5 µM), added for 15 min, 1 h, 6 h, and 18 h (for protein expression), 6 and 18 h (for gene expression), and 18 h (for migration studies). ERK inhibitor PD98059 (100 µM), p38 inhibitor SB239063 (20 µM), P2Y12 inhibitor MRS2395 (15 µM), and all histamine receptor antagonists were added 30 min before cell treatments (Table 1). At the end of incubations, the medium was removed and the cells were washed twice with PBS before collection. At these concentrations, none of the tested drugs interfered with microglial cell viability. In time-course experiments, controls at each time point were assessed and reported with value = 1, if found unchanged.

**Table 1:** Pharmacological compounds used in the study.

Drug	Concentration (μM)	Function
Orphenadrine	10	H1 antagonist
Ranitidine	10	H2 antagonist
Thioperamide	5	H3 antagonist
JNJ7777120	5	H4 antagonist
PD98059	100	ERK inhibitor
SB203580	20	p38 inhibitor
MRS2395	15	P2Y12 antagonist

### Antibodies

Antibodies for WB and IF were rabbit anti-HDC (1:500 WB; 1:200 IF, Abcam, USA); rabbit anti-HNMT (1:200 WB, IF, Atlas, Sweden); rabbit anti-DAO (1:200 WB and IF, Bioss, USA); rabbit anti-H1R (1:500 WB, 1:200 IF, Alomone, Israel); rabbit anti-H2R (1:500 WB and 1:200 IF, Alomone); rabbit anti-H3R (1:500 WB, 1:200 IF, Alomone); rabbit anti-H4R (1:500 WB, 1:200 IF, Santa Cruz); mouse anti-NOX2/gp91^phox^ (1:1,000 WB, BD Transduction Laboratories, USA); rabbit anti-phospho-NF-κB p65 (Ser536) (1:500 WB, CST, USA); rabbit anti-NF-κB p65 (1:500 WB, CST); rabbit anti-phospho-p44/42 MAPK (ERK1/2) (Thr202/Tyr204) (1:1,000 WB, CST); mouse anti-p44/42 MAPK (ERK1/2) (L34F12) (1:1,000 WB, CST); rabbit anti-phospho-p38 MAPK (Thr180/Tyr182) (1:500 WB, CST); mouse anti-p38 MAPK (A-12) (1:500 WB, Santa Cruz); rabbit anti-Arginase 1 (ARG1) (1:700 WB, Abcam); rabbit anti-P2Y12 (1:200 WB, Anaspec, USA); rat anti-CD11b (1:200 IF, AbD Serotec, USA); rabbit anti-Iba1 (1:500, WB, IF, Wako, USA); rat anti-CD68 (1:500, WB, IF, AbD Serotec); rabbit anti-CD163 (1:100 WB, Santa Cruz); rabbit anti-CD206 (1:500 WB, Abcam); mouse anti-GAPDH (1:2,500 WB, Calbiochem, USA).

### Western Blotting

Protein lysates were obtained by homogenization of mice cortex, lumbar spinal cord, and hypothalamus in homogenization buffer (20 mM HEPES, pH 7.4, 100 mM NaCl, 1% Triton X-100, 10 mM EDTA) added with protease inhibitor cocktail (Sigma-Aldrich). After sonication, lysates were kept on ice and centrifuged for 20 min at 14,000 × *g* at 4°C. Primary microglia were harvested in ice-cold RIPA buffer (PBS, 1% Nonidet P-40, 0.5% sodium deoxycholate, 0.1% SDS) added with protease inhibitor cocktail (Sigma-Aldrich). Lysates were kept on ice and then centrifuged for 10 min at 14,000 × *g* at 4°C. Supernatants were assayed for protein quantification with the Bradford detection kit (Bio-Rad Laboratories, Hercules, CA, USA). Proteins were separated by SDS-PAGE gels and transferred onto a nitrocellulose membrane (Amersham Biosciences, Cologno Monzese, Italy). Membranes were incubated with the specified antibodies overnight at 4°C and with HRP-conjugated secondary antibodies for 1 h and then detected using ECL Advance WB detection kit (Amersham Biosciences). Quantifications were performed by Kodak Image Station 440CF.

### IF and Confocal Analysis

Mice were anesthetized with chloral hydrate (500 mg/kg) and perfused intracardially with PBS. Spinal cords (L3–L5) were post-fixed overnight in 4% PFA, processed, and analyzed as described (22). Sections (30-µm thick) were incubated with the specified antibodies in PBS 0.3% Triton X-100 and 2% normal donkey serum at 4°C overnight, washed thoroughly, and incubated with appropriate fluorescent-conjugated secondary antibodies for 3 h at room temperature. The secondary antibodies used were Cy2-conjugated donkey anti-rabbit immunoglobulin G (IgG) (1:100, Jackson Immunoresearch, green IF) or Cy3-conjugated donkey anti-rat IgG (1:100, Alexa, Molecular Probes Inc., USA, red IF). PBS washes (3× 5 min) were performed and slides were cover slipped with Fluoromount medium (Sigma).

Microglial cultures were fixed for 20 min in 4% PFA and permeabilized for 5 min in PBS 0.1% Triton X-100. The cells were incubated overnight at 4°C in 1% BSA in PBS with the specified antibodies and then stained for 3 h with secondary antibodies. Nuclei were stained with 1 µg/ml Höechst 33258 for 5 min and cells were finally cover slipped with Fluoromount. The secondary antibodies used were Cy2-conjugated donkey anti-rabbit IgG (1:100, Jackson Immunoresearch) or Cy3-conjugated donkey anti-rat IgG (1:100, Alexa, Molecular Probes Inc.). Immunofluorescence was analyzed by a confocal laser scanning microscope (Zeiss, LSM700, Germany) equipped with four laser lines: 405, 488, 561, and 639 nm and using identical acquisition parameters for all the images. The brightness and contrast of the digital images were adjusted using the Zen software (Zeiss).

### Motility Assay

The microglia monolayer at approximately 95% confluence in serum-free medium was evaluated by scratch wound assay made on the cell plate with a P10 pipette tip (Gilson S.A.S., France). After pharmacological treatments, IF was performed with anti-CD11b and images taken by confocal laser scanning microscopy (Zeiss, LSM700, Germany) at 20× magnification, acquired using Zen Software (Zeiss), and analyzed by manual cell counting using NIH ImageJ Software. Cell motility was determined by counting the number of cells that migrated toward the middle of the wound within the 18-h period of treatment. Each experiment was carried out in triplicate.

### Quantitative Real-time Polymerase Chain Reaction

Total RNA was extracted with TRIZOL (Invitrogen) according to the manufacturer’s instruction and was quantified with the Nanodrop 100 System (Rockford, IL, USA) and the Agilent 2100 bioanalyzer (Santa Clara, CA, USA). QPCR for IL-6, IL-10, and IL-1β quantification was performed using SYBR green (Life Technologies) subsequent to reverse transcription using the Superscript Vilo cDNA Synthesis Kit (Life Technologies). Relative gene expression was calculated by ΔΔ*C*_t_ analysis relative to GAPDH expression levels. Primers used: GAPDH forward 5′-CATGGCCTTCCGTGTTTCCTA-3′; GAPDH reverse 5′-CCTGCTTCACCACCTTCTTGAT-3′; IL-6 forward 5′-GAGGATACCACTCCCAACAGACC-3′; IL-6 reverse 5′-AAGTGCATCATCGTTGTTCATACA-3′; IL-10 forward 5′-GCATGGCCCAGAAATCAAGG-3′; IL-10 reverse 5′-GAGAAATCGATGACAGCGCC-3′; IL-1β forward 5′-GCAACTGTTCCTGAACTCAACT-3′; IL-1β reverse 5′-ATCTTTTGGGGTCCGTCAACT-3′.

### Identification and Functional Characterization of Histamine-Related Genes in sALS Patients

Whole-genome expression profile analyses of motor cortex from control (10) and sALS patients (31) has allowed to distinguish sALS patients into two gene expression-based subgroups (sALS1 and sALS2) each associated with differentially expressed genes and pathways (23). Transcriptome data are available from the ArrayExpress database with the accession number E-MTAB-2325. Here, we filtered this dataset by focusing on genes implicated in histamine cascades, by using histamine metabolism/signaling query keywords in Gene Ontology database (http://www.geneontology.org/). In addition, to provide a more comprehensive characterization of the histamine-related events occurring in sALS pathology, we investigated the expression changes of this filtered gene list also in the spinal cord samples obtained from the same patient cohort. Fold-change values for sALS patients/control were calculated and converted into negative reciprocal, if the fold-change was less than 1. Statistical assessment of gene expression data was performed by using GeneSpring GX v13.1 software package (Agilent Technologies, USA). Genes with a corrected *p* value <0.05 (one-way ANOVA followed by the Benjamin–Hochberg False Discovery Rate and the Tukey’s *Post Hoc* test) were considered statistically significant. To analyze gene expression changes of histamine-related candidates in the context of known biological pathways, we used the private MetaCore (Genego) repository of functional annotations (24).

### Data Analysis

Data are presented as mean ± SEM. Analysis was performed using GraphPad Prism 7.03 software (GraphPad Software, San Diego, CA, USA). Statistical differences over two groups were verified by Student’s *t*-test or by the Mann–Whitney rank sum test when a non-normal distribution of the data is assumed. For multiple comparisons, differences were analyzed by one-way analysis of variance ANOVA followed by *Post Hoc* Tukey’s test, A probability level of 0.05 or less was considered to be statistically significant.

## Results

### Histamine Receptors and Metabolizing Enzymes Are Expressed and Modulated in SOD1-G93A Primary Microglia

**Figure 1:**
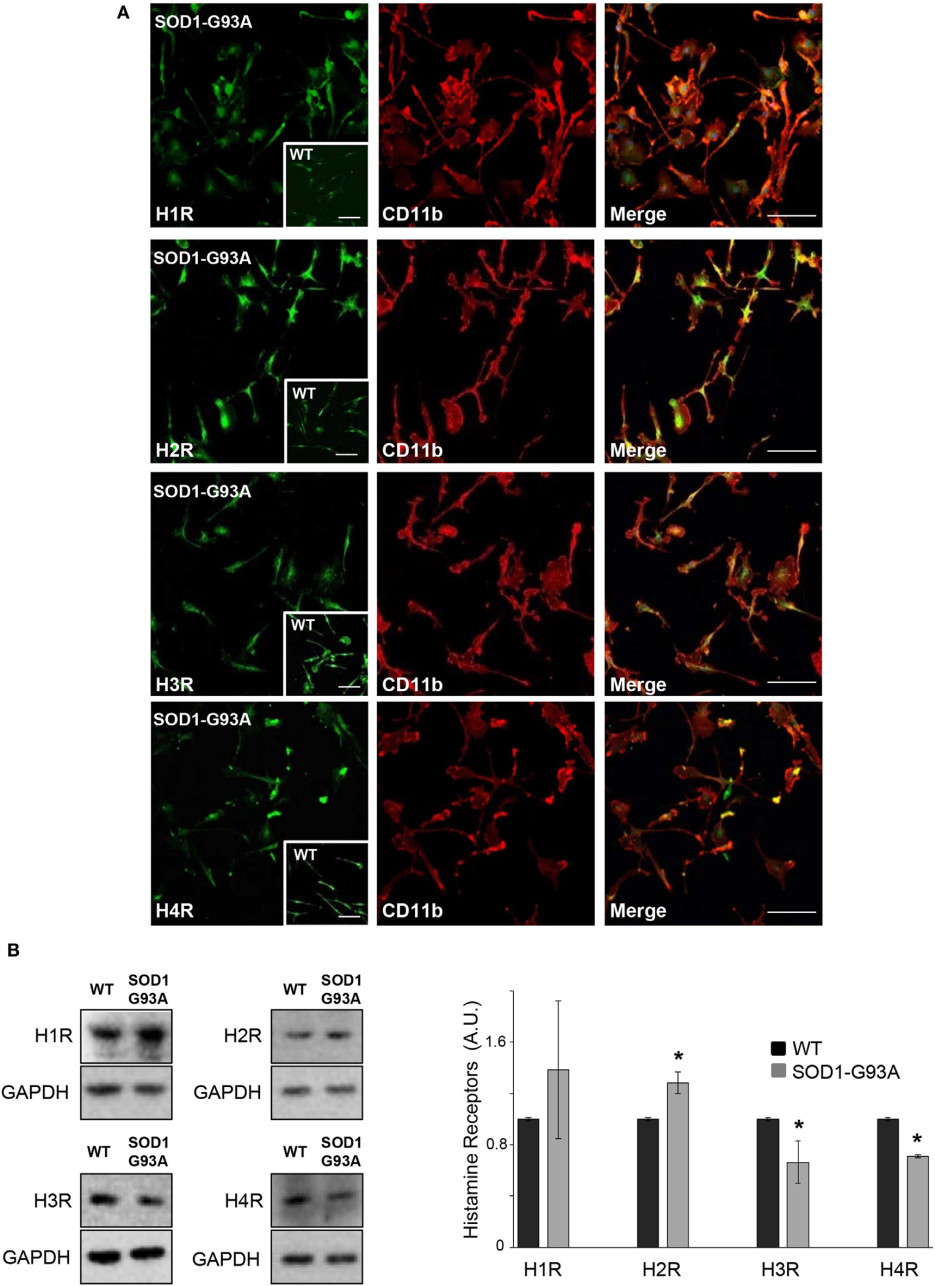
SOD1-G93A microglia express histaminergic receptors. **(A)** Primary microglia from SOD1-G93A mice were stained with anti-CD11b (red) and anti-H1R, anti-H2R, anti-H3R, and anti-H4R (green). Höechst 33258 was used for nuclei (scale bar 50 µm). In inserts, wild-type (WT) microglia (scale bar 50 µm). **(B)** Equal amounts of WT and SOD1-G93A primary microglia total lysates were subjected to western blotting with anti-H1R, anti-H2R, anti-H3R, and anti-H4R. Anti-GAPDH was used for protein normalization. Data represent mean ± SEM of *n* = 3 independent experiments. Statistical significance was calculated by Student’s *t*-test, as referred to WT cells, *p < 0.05.

**Figure 2:**
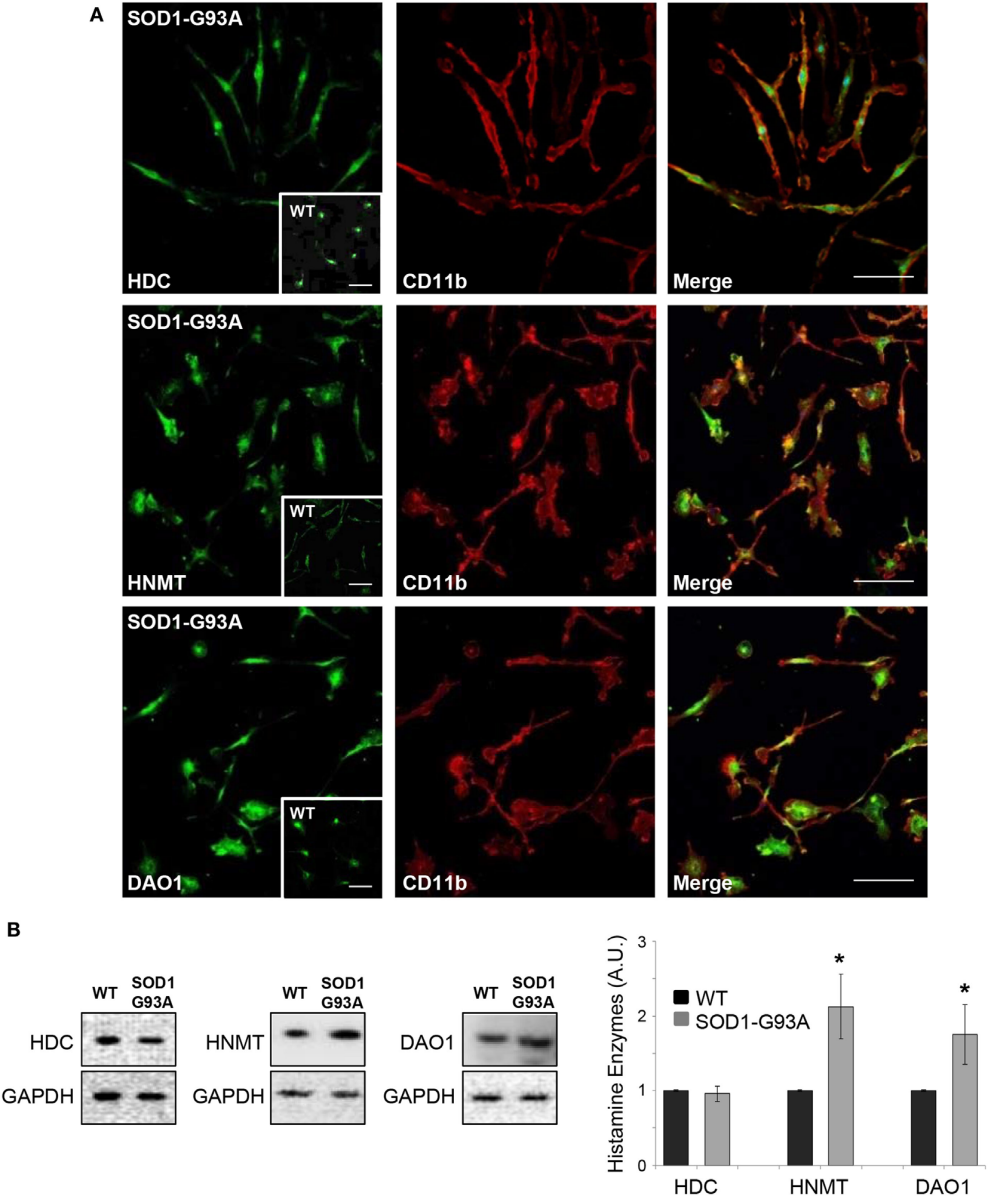
SOD1-G93A microglia express histaminergic enzymes. **(A)** Microglia from SOD1-G93A mice were stained with anti-CD11b (red) and anti-histidine decarboxylase (HDC), anti-histamine *N*-methyltransferase (HNMT), and anti-diamine oxidase (DAO) (green). Höechst 33258 was used for nuclei (scale bar 50 µm). In inserts, wild-type (WT) microglia (scale bar 50 µm). **(B)** Equal amounts of WT and SOD1-G93A primary microglia total lysates were subjected to western blotting with anti-HDC, anti-HNMT, and anti-DAO. Anti-GAPDH was used for protein normalization. Data represent mean ± SEM of *n* = 3 independent experiments. Statistical significance was calculated by Student’s *t*-test, as referred to WT cells, *p < 0.05.

We used primary microglia cultures from SOD1-G93A mice to explore the histaminergic system in ALS. We first mapped protein expression of histamine receptors and metabolizing enzymes in SOD1-G93A microglia compared to WT. As shown by confocal analysis, H1R-H4R (Figure 1A), HDC, HNMT, and DAO (Figure 2A) were uniformly distributed in the majority of SOD1-G93A microglia (as well as WT microglia, see inserts), co-localizing with CD11b specific microglia marker. Moreover, statistically significant higher expression of H2R and lower expression of H3R and H4R was demonstrated by WB in SOD1-G93A with respect to WT microglia (Figure 1B), together with significant upregulation of HNMT and DAO, but not of HDC, in SOD1-G93A compared to WT (Figure 2B).

### MAPKs Pathway Is Activated by Histamine in SOD1-G93A Microglia

**Figure 3:**
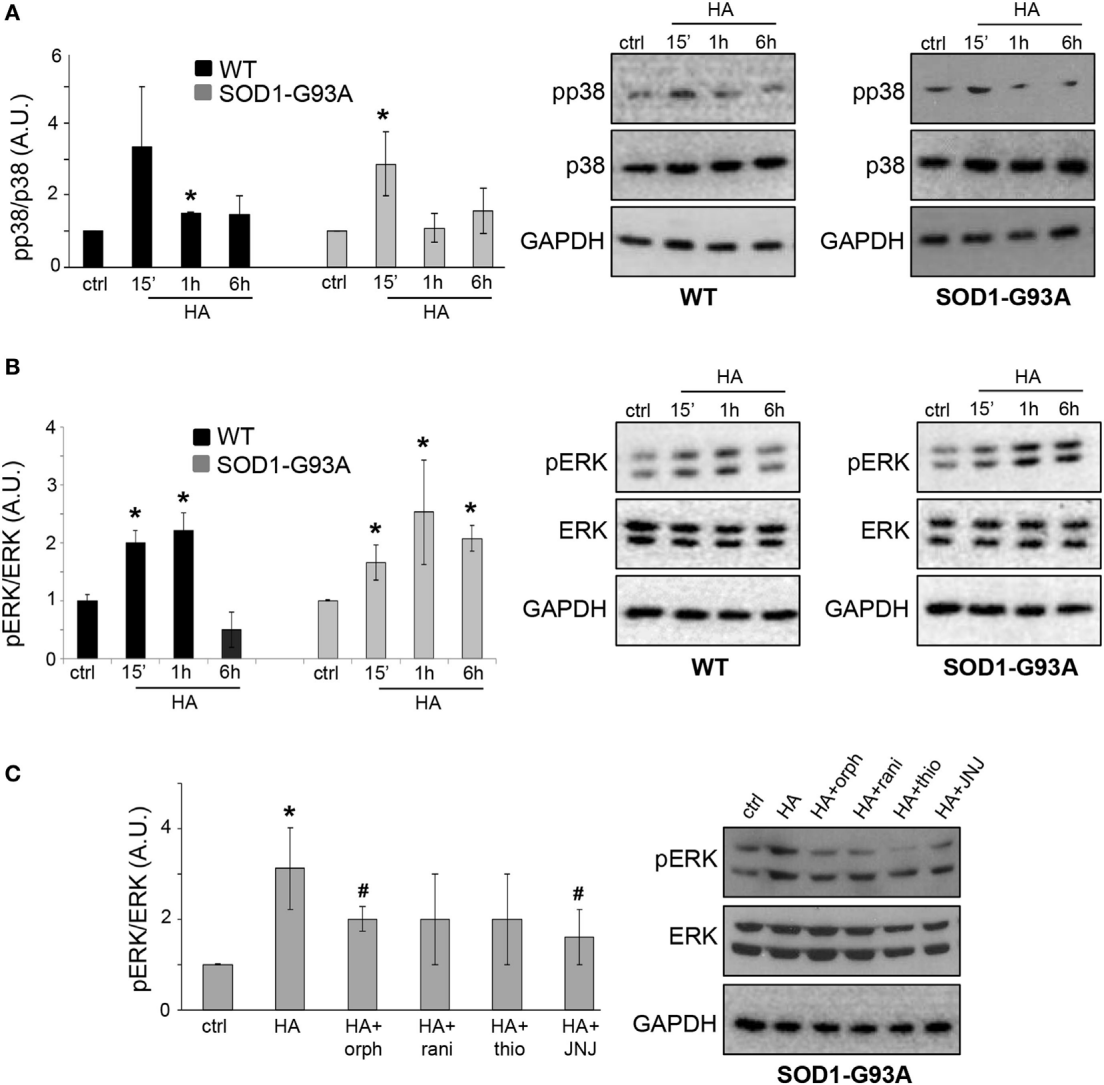
MAPKs are activated by histamine in SOD1-G93A microglia. Wild-type (WT) and SOD1-G93A primary microglia were treated with histamine (100 µM) for 15 min–1 h–6 h and equal amounts of total lysates were subjected to SDS-PAGE, western blotting, and immunoreactions with anti-p-p38 and anti-p-38 **(A)** or with anti-pERK and anti-ERK **(B)**. **(C)** SOD1-G93A microglia stimulated with histamine (100 µM) for 1 h in the presence of specific antagonists for H1R (orphenadrine, 10 µM), H2R (ranitidine, 10 µM), H3R (thioperamide, 5 µM), or H4R (JNJ-7777120, 5 µM) were subjected to immunoreactions with anti-pERK and anti-ERK. Anti-GAPDH was used for protein normalization. HA, histamine. Data represent mean ± SEM of *n* = 4 independent experiments. Statistical significance was calculated by ANOVA followed by *Post Hoc* Tukey’s test, as referred to ctrl cells, *p < 0.05 or to histamine-treated cells, ^#^*p* < 0.05.

Previous reports demonstrated that histamine modulates the microglial MAPK pathway (25). To dissect the involvement of phosphorylation-dependent events in the signal transduction mechanisms downstream of histamine receptor activation in ALS microglia, we investigated the phosphorylation of p38 and ERK1/2. Histamine induced a transient phosphorylation of p38 in both WT and SOD1-G93A microglia (Figure 3A). The phosphorylation of ERK1/2 induced by histamine instead persisted for up to 6 h only in SOD1-G93A respect to WT microglia (Figure 3B). Controls at each time point were assessed and found unchanged (data not shown). In WT microglia, the upregulation of p-ERK1/2 after 1 h of histamine treatment was inhibited by H1R and H4R antagonists (data not shown). In SOD1-G93A microglia, while H1R-H4R antagonists alone failed to significantly affect the levels of p-ERK1/2 (ctrl: 1 ± 0.01; orphenadrine: 1.3 ± 0.1; ranitidine: 1.7 ± 0.9; thioperamide: 1.4 ± 0.3; JNJ7777120: 1.6 ± 0.4; *p* > 0.05), the histamine-dependent ERK1/2 phosphorylation was significantly prevented by H1R and H4R antagonists (Figure 3C).

### Histamine Affects NF-κB Pathway in SOD1-G93A Microglia

**Figure 4:**
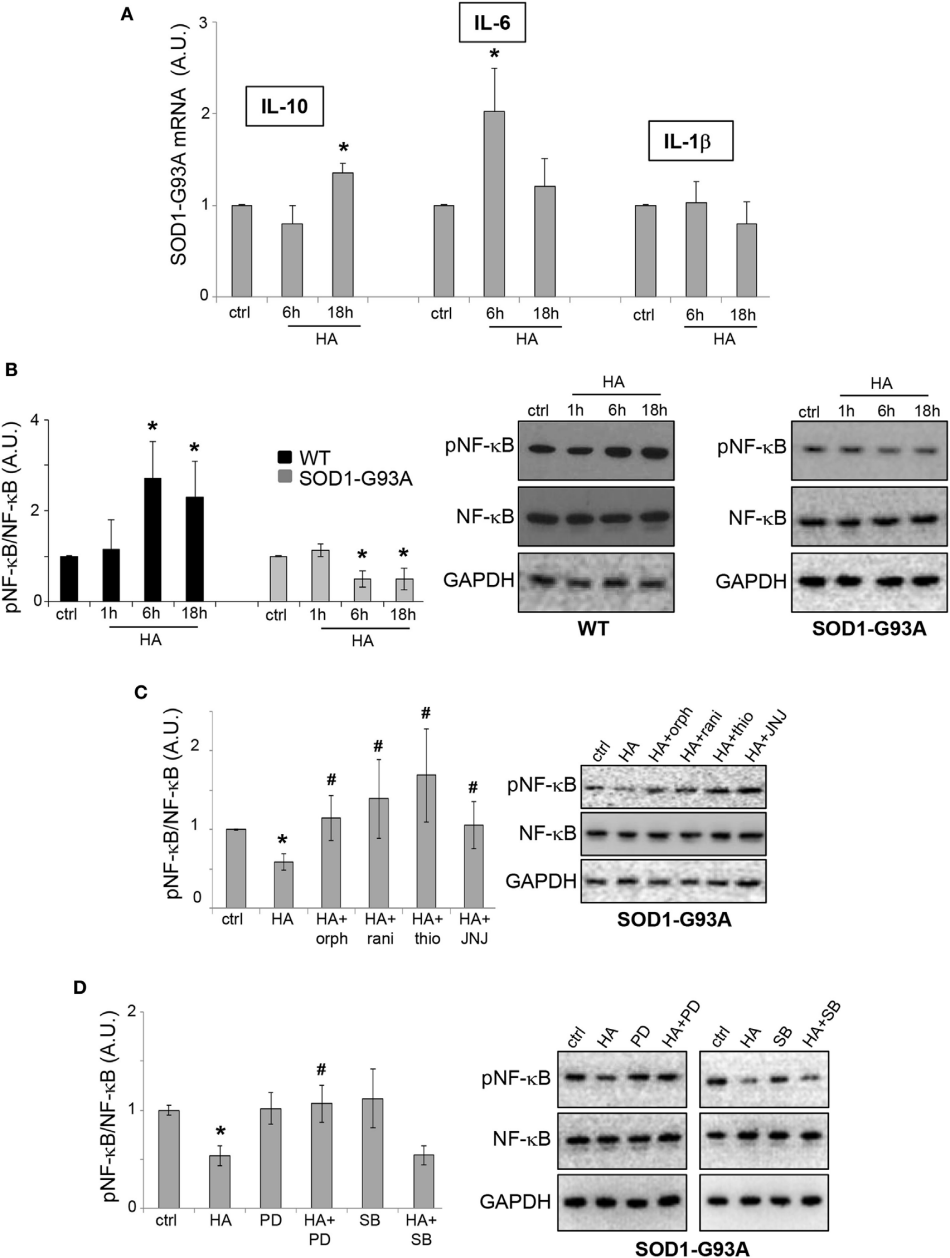
NF-κB pathway is modulated by histamine in SOD1-G93A microglia. **(A)** Total RNA was extracted from SOD1-G93A microglia stimulated with histamine (100 µM) for 6–18 h and the expression profiles of IL-10, IL-6, and IL-1β were examined by qRT-PCR. **(B)** Wild-type (WT) and SOD1-G93A microglia exposed to 100 µM histamine for 1–6–18 h were subjected to immunoreactions with anti-pNF-κB and anti-NF-κB. **(C)** SOD1-G93A microglia stimulated with histamine (100 µM) for 6 h in the presence of H1R–H2R–H3R–H4R antagonists were subjected to immunoreactions with anti-pNF-κB and anti-NF-κB. **(D)** SOD1-G93A microglia treated with histamine (100 µM) for 6 h in the presence of specific inhibitors of pERK (PD98059, 100 µM) and pp38 (SB203580, 20 µM) were subjected to immunoreactions with anti-pNF-κB and anti-NF-κB. Anti-GAPDH was used for protein normalization. HA, histamine. Data represent mean ± SEM of *n* = 4 independent experiments. Statistical significance was calculated by ANOVA followed by *Post Hoc* Tukey’s test, as referred to ctrl cells, *p < 0.05 or to histamine-treated cells, ^#^*p* < 0.05.

NF-κB is a signaling pathway known to be directly modulated by histamine in microglia (25). Moreover, NF-κB is involved in driving gene expression of pro-inflammatory and anti-inflammatory cytokines, enzymes, and adhesion molecules, many of which are tightly regulated in ALS (26). In particular, IL-6, IL-10, and IL-1β are among the cytokines directly regulated by NF-κB, and we previously demonstrated that IL-6 is downregulated in SOD1-G93A as compared to WT microglia (27). Here, we proved that histamine upregulated IL-6 and IL-10 mRNA expression in SOD1-G93A microglia, respectively, after 6 and 18 h, although not modifying the levels of IL-1β (Figure 4A). Furthermore, we showed that while histamine increased pNF-κB in WT microglia, it induced a significant downregulation of pNF-κB in SOD1-G93A microglia (Figure 4B). In WT microglia, the upregulation of pNF-κB was inhibited by H1R, H3R, and H4R antagonists (data not shown). In SOD1-G93A microglia, while histamine antagonists H1R-H4R alone failed to significantly affect the levels of pNF-κB respect to ctrl cells (ctrl: 1 ± 0.01; orphenadrine: 0.9 ± 0.2; ranitidine: 0.7 ± 0.1; thioperamide: 1.1 ± 0.3; JNJ7777120: 1.1 ± 0.3; *p* > 0.05), the effect of histamine on pNF-κB was prevented by the selective H1R-H4R antagonists orphenadrine, ranitidine, thioperamide, and JNJ7777120 (Figure 4C). Moreover, the inhibitory effect of histamine on pNF-κB was prevented by the ERK inhibitor PD98059, but not by the p38 inhibitor SB203580 (Figure 4D), thus reinforcing the role of ERK kinase in the histamine signaling of SOD1-G93A microglia.

### Histamine Modulates Inflammatory Markers and Induces SOD1-G93A Microglia Migration

**Figure 5:**
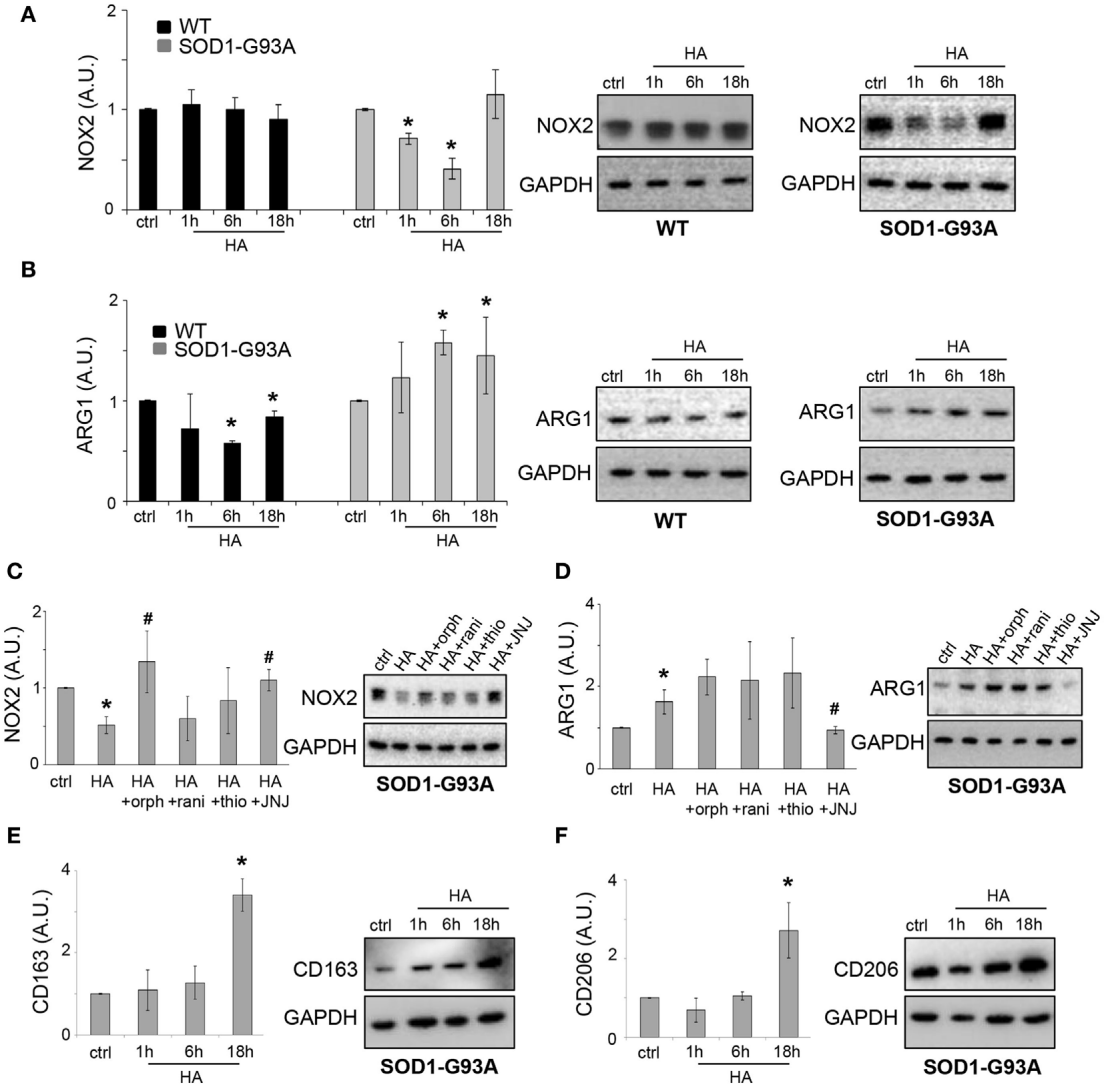
Histamine modulates inflammation in SOD1-G93A microglia. Wild-type (WT) and SOD1-G93A primary microglia were exposed to 100 µM histamine for 1–6–18 h and equal amounts of total lysates subjected to western blotting (WB) and immunoreactions with anti-NADPH oxidase 2 (NOX2) **(A)**, anti-ARG-1 **(B)**. SOD1-G93A microglia stimulated with histamine (100 µM) in the presence of H1R–H2R–H3R–H4R antagonists for 6 h were subjected to immunoreactions with anti-NOX2 **(C)** or anti-ARG-1 **(D)**. SOD1-G93A primary microglia were exposed to 100 µM histamine for 1–6–18 h and equal amounts of total lysates subjected to WB and immunoreactions with anti-CD163 **(E)** or anti-CD206 **(F)**. Anti-GAPDH was used for protein normalization. HA, histamine. Data represent mean ± SEM of *n* = 4 independent experiments. Statistical significance was calculated by ANOVA followed by *Post Hoc* Tukey’s test, as referred to ctrl cells, *p < 0.05 or to histamine-treated cells, ^#^*p* < 0.05.

In addition to NF-κB, we demonstrated that histamine inhibited the protein expression of pro-inflammatory NADPH oxidase 2 (NOX2) in SOD1-G93A, but not WT microglia (Figure 5A).

As shown in Figure 5C, in SOD1 microglia, the inhibitory effect of histamine on NOX2 was dependent on H1R and H4R, being prevented by the selective H1R and H4R antagonists orphenadrine and JNJ7777120. Histamine receptor antagonists alone failed to affect the levels of NOX2 in SOD1-G93A microglia respect to ctrl cells (ctrl: 1 ± 0.01; orphenadrine: 1.2 ± 0.4; ranitidine: 0.7 ± 0.2; thioperamide: 0.7 ± 0.4; JNJ7777120: 1.1 ± 0.4; *p* > 0.05).

The anti-inflammatory marker ARG1 was instead increased by histamine in SOD1-G93A but inhibited in WT microglia (Figure 5B). In WT microglia, the downregulation of ARG1 was inhibited by H3R and H4R antagonists (data not shown). In SOD1-G93A microglia, while histamine receptor antagonists alone failed to significantly affect the levels of ARG1 respect to ctrl cells (ctrl: 1 ± 0.01; orphenadrine: 2.2 ± 0.7; ranitidine: 2.9 ± 0.6; thioperamide: 1.3 ± 0.5; JNJ7777120: 1.4 ± 0.4; *p* > 0.05), the effect of histamine on ARG1 was dependent on the activation of H4R, being prevented only by the selective antagonist JNJ7777120 (Figure 5D). Finally, we demonstrated that, in SOD1-G93A microglia, histamine after 18 h of treatment increased the levels of other two well-known (28) anti-inflammatory phenotypic markers such as CD163 (Figure 5E) and CD206 (Figure 5F).

**Figure 6:**
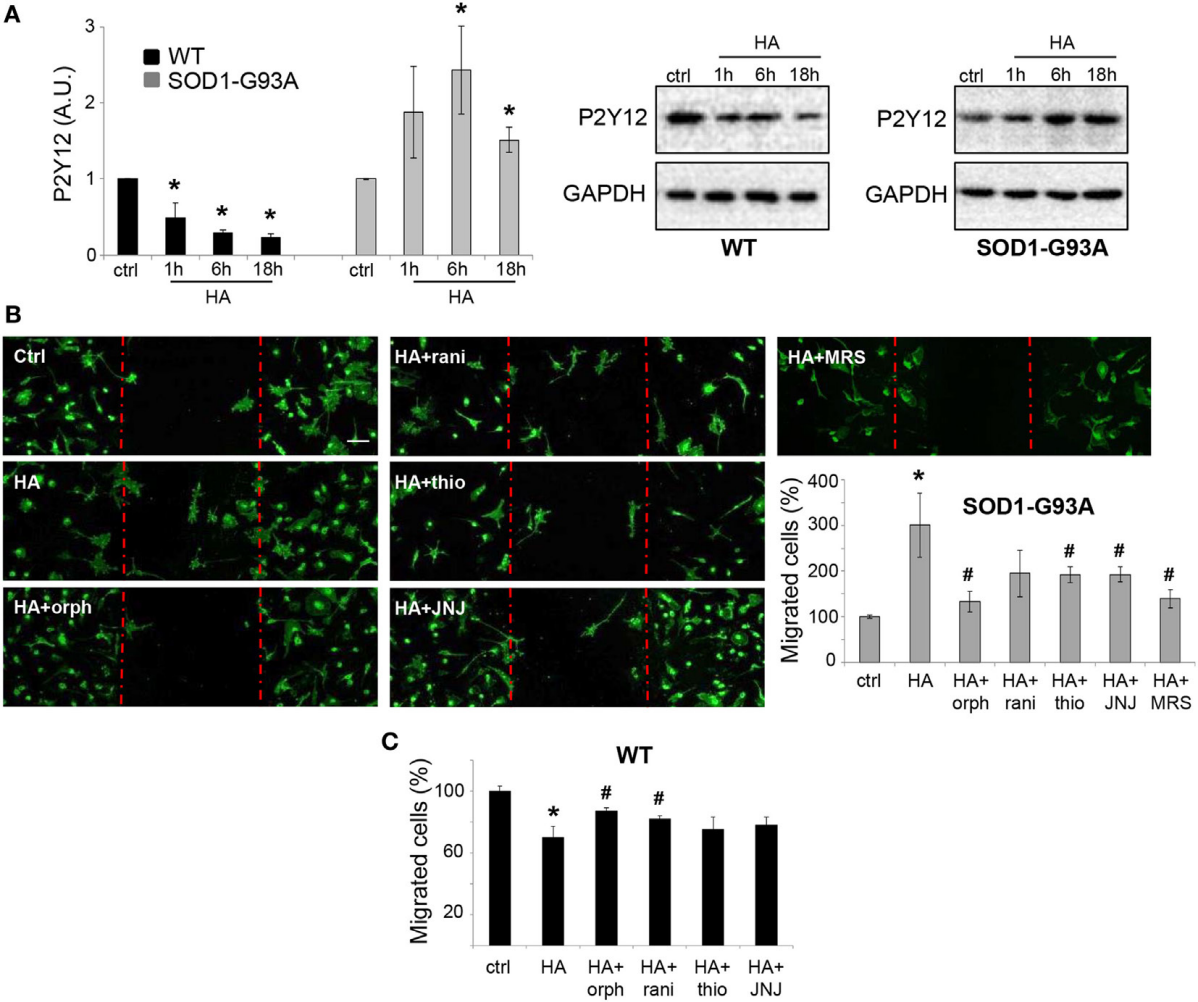
Histamine induces migration in SOD1-G93A microglia. **(A)** Wild-type (WT) and SOD1-G93A primary microglia were exposed to 100 µM histamine for 1–6–18 h and equal amounts of total lysates subjected to western blotting and immunoreactions with anti-P2Y12. Anti-GAPDH was used for protein normalization. **(B)** SOD1-G93A microglia were stimulated with 100 µM histamine in the presence or absence of H1R–H2R–H3R–H4R antagonists or P2Y12 antagonist (MRS2395, 10 µM) for 18 h and stained with anti-CD11b (green). Scale bar 50 µm. The number of migrating cells was then quantified. **(C)** WT microglia were stimulated with 100 microM histamine in the presence or absence of H1R-H2R-H3R-H4R antagonists for 18 h and the number of migrating cells was quantified, HA, histamine. Data represent mean ± SEM of *n* = 3 independent experiments. Statistical significance was calculated by ANOVA followed by *Post Hoc* Tukey’s test, as referred to ctrl cells, *p < 0.05 or to histamine-treated cells, ^#^*p* < 0.05.

By analyzing the P2Y12 purinergic receptor expression known to be downregulated in SOD1-G93A microglia and ALS disease progression (29, 30), we showed that similar to the other anti-inflammatory markers, also P2Y12 receptor was significantly decreased by histamine in WT, but increased in SOD1-G93A microglia (Figure 6A).

Since it was previously reported that histamine affects microglia migration (10), we performed the wound-healing scratch assay to evaluate histamine on SOD1-G93A microglia motility. As shown by IF with CD11b marker, histamine significantly enhanced microglia migration after 18 h (Figure 6B) and the effect was dependent on H1R, H3R, and H4R, being significantly reduced by orphenadrine, thioperamide, and JNJ7777120, respectively (Figure 6B). Because P2Y12 receptor is among the signaling pathways controlling microglia migration (31, 32), we finally demonstrated that histamine-induced SOD1-G93A migration was dependent on P2Y12, receptor being inhibited by the specific inhibitor MRS2395 (Figure 6B). Antagonists alone failed to significantly affect SOD1-G93A microglia migration (data not shown). Conversely to what occurs in SOD1-G93A microglia, in WT microglia histamine inhibited cell migration and this effect was dependent on H1R and H2R (Figure 6C).

Overall, these results suggest that in the SOD1-G93A context histamine polarizes microglia toward an anti-inflammatory phenotype.

Because histamine cannot enter the blood–brain barrier, we injected a single dose of its precursor histidine in SOD1-G93A mice at 140 days of age and investigated inflammatory parameters. We demonstrated a decrease in Iba1- and CD68-positive microglia/macrophages in the lumbar spinal cord of histidine-treated mice (Figure S1A in Supplementary Material), concomitantly with a significant increase of anti-inflammatory ARG1 and a decrease of pro-inflammatory NOX2 and NF-κB (Figure S1B in Supplementary Material).

### Histamine Receptors and Enzymes Are Dysregulated in Cortex, Spinal Cord, and Hypothalamus of SOD1-G93A Mice during Disease Progression

**Figure 7:**
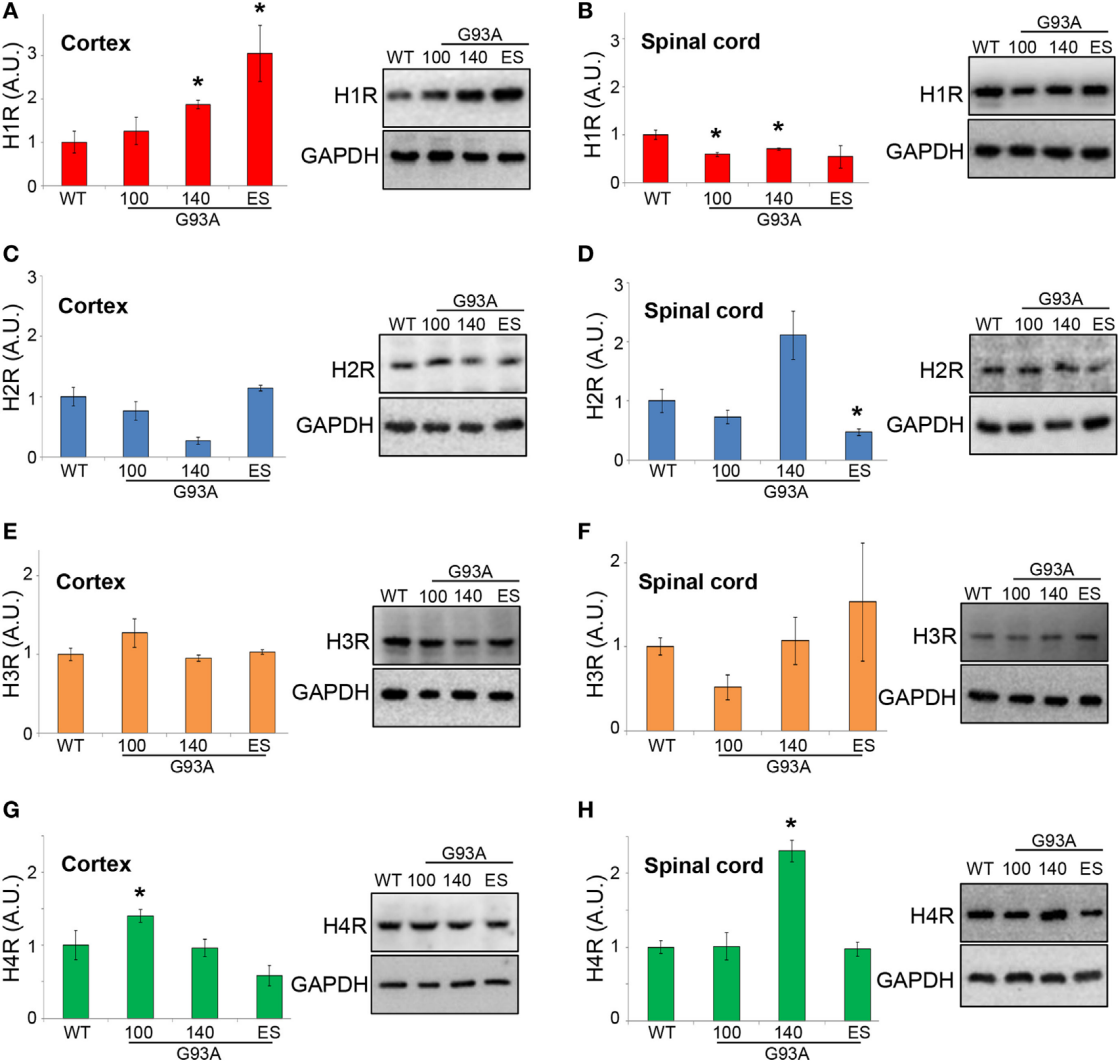
Protein expression levels of H1-H4R in cortex and lumbar spinal cord from wild-type (WT) and SOD1-G93A mice. Cortex **(A,C,E,G)** and lumbar spinal cord **(B,D,F,H)** from WT (~20 weeks) and SOD1-G93A mice at different stages of disease (100, 140 days, and end stage) (*n* = 4/group) were subjected to SDS-PAGE, western blotting, and immunoreactions with anti-H1R, anti-H2R, anti-H3R, and anti-H4R. Anti-GAPDH was used for protein normalization. Data represent mean ± SEM. Statistical significance was calculated by ANOVA followed by *Post Hoc* Tukey’s test, as referred to WT mice, *p < 0.05.

To establish if the histaminergic system is dysregulated in SOD1-G93A mice, we evaluated by WB analysis the protein levels of H1R–H4R and HDC, HNMT, and DAO in cortex and lumbar spinal cord of SOD1-G93A mice at different phases of the disease, i.e., at presymptomatic (100 days), symptomatic (140 days), and end stage, compared to WT mice. H1R was upregulated in the cortex at symptomatic and end stage of the disease (Figure 7A), while downregulated in the spinal cord at 100 and 140 days (Figure 7B). H2R was decreased only at symptomatic phase (Figure 7C) in the cortex, and only at end stage in the spinal cord (Figure 7D). H3R was not modulated during the course of the disease (Figures 7E,F), whereas H4R was upregulated at presymptomatic phase in the cortex (Figure 7G), and at symptomatic phase in the spinal cord (Figure 7H).

**Figure 8:**
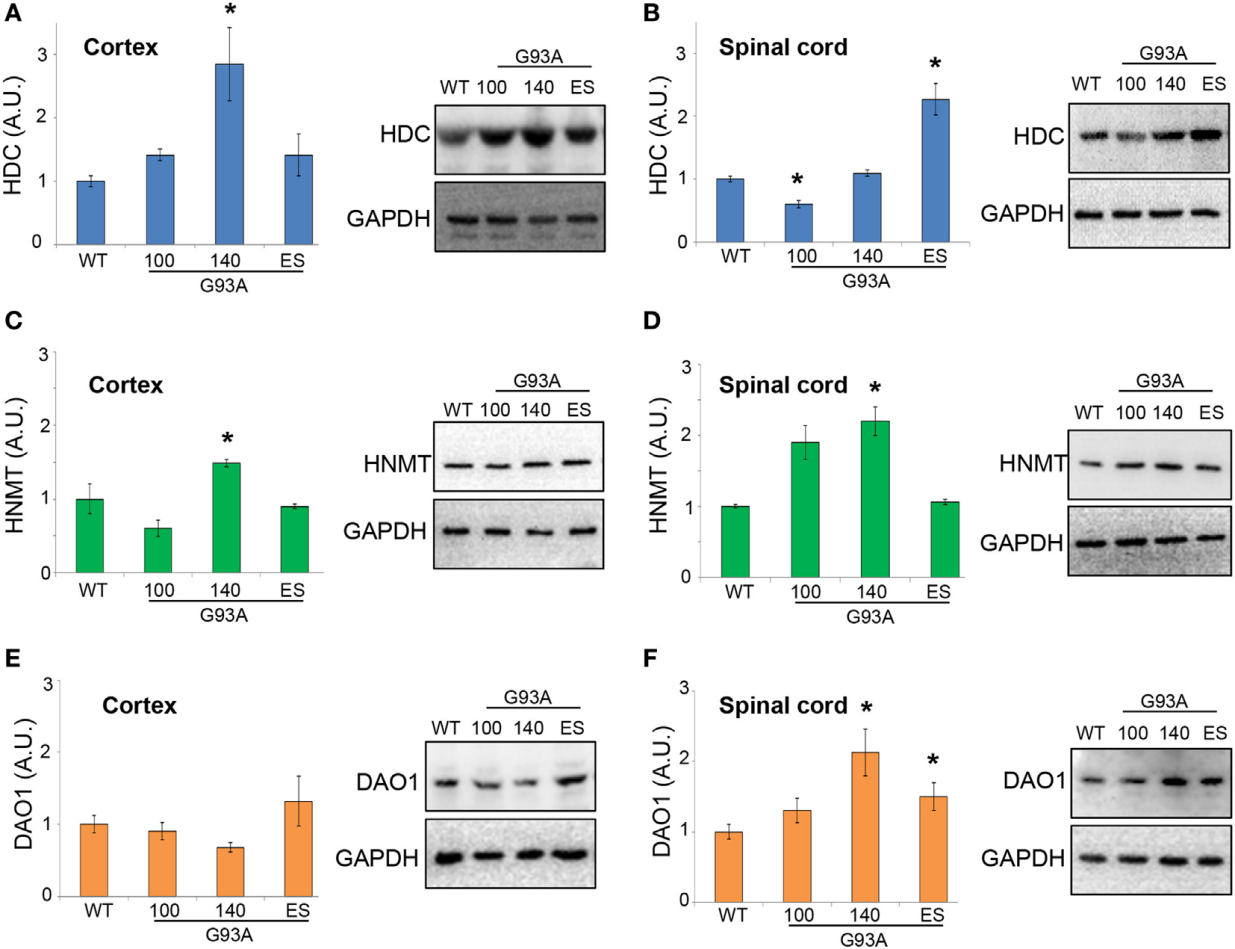
Protein expression levels of histidine decarboxylase (HDC), histamine *N*-methyltransferase (HNMT), and diamine oxidase (DAO) in cortex and lumbar spinal cord from wild-type (WT) and SOD1-G93A mice. Cortex **(A,C,E)** and lumbar spinal cord **(B,D,F)** from WT (~20 weeks) and SOD1-G93A mice at different stages of the disease (100, 140 days, and end stage) (*n* = 4/group) were subjected to SDS-PAGE, western blotting, and immunoreactions with anti-HDC, anti-HNMT, and anti-DAO. Anti-GAPDH was used for protein normalization. Data represent mean ± SEM. Statistical significance was calculated by ANOVA followed by *Post Hoc* Tukey’s test, as referred to WT mice, *p < 0.05.

Moreover, in SOD1-G93A mice, we demonstrated that HDC was significantly upregulated in the cortex at 140 days of age (Figure 8A), while in lumbar spinal cord we observed a significant downregulation at 100 days and an increase at end stage of the disease (Figure 8B). HNMT was significantly upregulated in both cortex (Figure 8C) and lumbar spinal cord (Figure 8D) at 140 days of age. DAO was not significantly affected during the course of the disease in the cortex (Figure 8E), but upregulated in the spinal cord of SOD1-G93A mice at symptomatic and end stage of disease (Figure 8F). The majority of histamine in the brain is produced in the tuberomamillary nucleus in the posterior hypothalamus (1). For this reason, we finally analyzed this region and observed, by WB analysis, a significant upregulation of H1R and H3R at end stage of the disease and of HDC, DAO, and HNMT at symptomatic phases of the disease (Figures S2A–G in Supplementary Material).

### Dysregulated Expression of Histamine-Related Genes in Cortex and Spinal Cord of Sporadic ALS Patients

**Figure 9:**
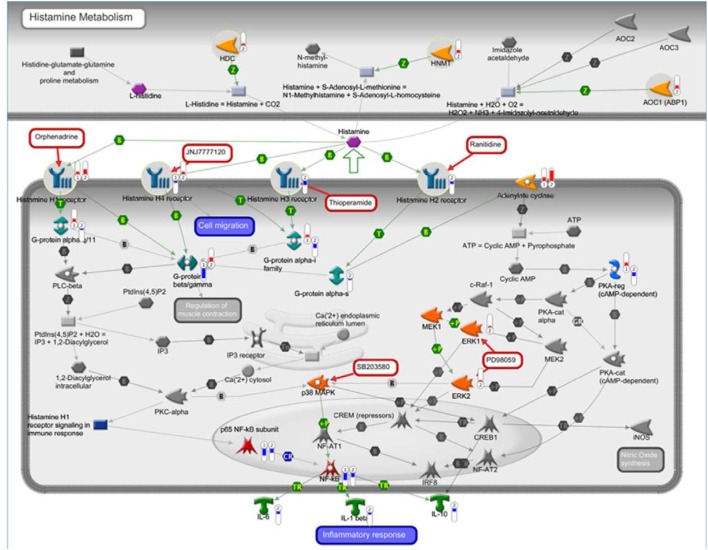
Histaminergic-related potential candidate genes for amyotrophic lateral sclerosis (ALS). The interaction pathway map represents histamine-related genes differentially deregulated in cortex of two subgroups of sporadic ALS (sALS) patients (sALS1 and sALS2). The map was created using MetaCore Pathway Map Creator tool (GeneGo). Gene expression values are presented on the map as “thermometer-like” figures (red for upregulation, blue for downregulation, and thermometer height is relative to fold-change) with sALS1 patients’ data represented as thermometer #1, and sALS2 patients as thermometer #2.

Recently, our group characterized the transcription profile of 41 motor cortex samples from 31 sALS patients and 10 neurologically healthy individuals. By performing unsupervised hierarchical clustering, we differentiated controls from sALS patients and segregated these latter into two clusters (sALS1 and sALS2), each categorized by differentially expressed genes and pathways (23). To explore the potential involvement of the histaminergic system in ALS patients, we examined our genomic data of cortex and spinal cord from sALS patients for expression of genes implicated in histamine signaling and metabolism. Our analysis revealed 98 histamine-related genes differentially deregulated in the cortex (Figure 9; Table 2) and spinal cord (Table 2) of the two subgroups of sALS patients versus control. Among deregulated genes, we observed the selective and statistically significant increased expression of three genes involved in the metabolism of histamine, HDC, HNMT, and DAO (AOC1), in the cortex of sALS2 patients. Moreover, statistically significant increased expression of the gene encoding histamine H1R was observed in the cortex of both sALS clusters, while expression of H2R and H3R was selectively reduced in sALS2 patients. H4R was differently modulated in sALS1 (downregulated) and in sALS2 (upregulated) respect to control patients. Spinal cord analysis showed deregulation of *DAO, H1R*, and *H3R* in sALS patients (Table 2).

**Table 2:** Fold-change differential expression of histamine-related genes in motor cortex and spinal cord of sporadic ALS (sALS) patients.

Gene symbol	Motor cortex	Motor cortex	Spinal cord	Spinal cord

	sALS1/control	sALS2/control	sALS1/control	sALS2/control
ADCY1	–	2.43	–	–
ADCY2	3.11	–	–	1.66
ADCY3	–	−3.57	–	–
ADCY6	1.85	–	–	–
ADCY9	–	9.54	–	2.87
AOC1	–	2.79	–	1.54
CREB1	–	9.41	–	2.75
CREM	1.64	−1.65	–	–
GNA11	2.08	–	–	–
GNAI1	–	−2.25	–	–
GNAOl	1.98	−3.87	2.09	–
GNAQ	2.51	−0.54	–	–
GNAS	–	−0.35	−1.75	−1.73
GNAZ	–	−2.15	–	–
GNB1	–	−3.53	–	–
GNB2	1.93	2.35	–	–
GNB4	–	−2.29	−1.57	–
GNG10	–	−2.95	−1.51	–
GNG11	–	−2.93	−1.66	–
GNG13	–	−2.95	–	−1.59
GNG3	3.42	2.97	4.55	2.65
GNG4	–	−2.21	–	–
GNG5	1.85	−1.72	–	–
GNG7	–	−3.13	–	–
GNGT1	−9.24	3.91	2.86	1.56
HDC	–	1.51	–	–
HNMT	–	2.62	–	–
HRH1	1.96	4.20	−1.37	−3.37
HRH2	–	−1.74	–	–
HRH3	–	−3.46	3.31	3.24
HRH4	−1.51	2.03	–	–
ICAM1	–	1.27	–	–
IL10	–	−2.72	–	
IL12B	–	1.93		
IL1B	–	−1.63		
IL6	–	−2.23		−1.57
ITPR1	–	−5.07		−1.55
MAP2K2	–	−2.35		
MAPK1	–	0.05		
MMP1	–	3.10		
MMP13	–	7.53		
MMP9	–	−3.45		
NFATC1	1.94	–		
NFATC2	−2.45	–	–	−1.66
NFKB1	–	−3.01	−1.61	−1.67
NFKB2	−1.48	–		–
NOS2	–	−2.44		
PLCB1	–	−5.76		
PLCB3	–	4.65	1.53	
PRKACA	–	−0.36		
PRKACB	–	−4.34		
PRKAR1A	2.32	−3.24		−1.81
PRKAR2A	–	−0.07		−1.51
PRKAR2B	–	−1.81		
PRKCA	–	−0.97		
RAF1	–	−1.40		
REL	1.93	2.90		
RELA	−6.45	−3.83	–	−1.71
RELB	1.84	–		
TNF	−1.43	–		
VCAM1	–	3.93	–	–

*The table lists the principal histamine-related genes differentially expressed in motor cortex and spinal cord of sALS patients (subgroups sALS1 and sALS2) in comparison with controls. Expression levels are indicated as fold-change values*.

## Discussion

Activation of microglia is a complex process that promotes numerous different phenotypes (33) and, particularly in ALS pathogenesis, microglia is known to proliferate and switch their activated phenotype from protective anti-inflammatory to neurotoxic pro-inflammatory (34, 35). The main focus of this study was thus to investigate if and how histamine might prime SOD1-G93A microglia. In particular, our hypothesis is that histamine should be able to counteract the pro-inflammatory phenotype of SOD1-G93A microglia, if indeed histamine exerts in ALS an anti-inflammatory role as previously demonstrated in other neurodegenerative diseases (6). This seems to be the case, having we proved that histamine reduced pro-inflammatory NOX2 and NF-κB expression *via* ERK signaling, and increased anti-inflammatory IL-6, IL-10, ARG1, P2Y12, CD163, and CD206, particularly by acting through H1R and H4R. On this matter, previous studies reported that NOX2 activates the NF-κB system in ALS microglia (36) in turn upregulating NOX2 (37), and that inactivation of NOX2 decreases reactive oxygen species production and extends survival of SOD1-G93A mice, thus proposing NOX2 as a gene modifier in ALS (38). By acting on both NOX2 and NF-κB signaling, histamine might thus exert its beneficial role in SOD1-G93A microglia by modulating the cross talk between oxidative stress and neuroinflammatory mechanisms. ERK kinases are known to trigger the nuclear accumulation and activity of NF-κB and additional transcription factors which in turn can modulate cytokines and inflammatory mediators’ expression in monocytes, macrophages, and microglia (39). Moreover, ERKs can behave as anti-inflammatory signals that suppress the expression of NF-κB (40). With respect to histamine, it seems that ERK kinases activation might be a condition necessary for inhibition of NF-κB. This would suggest that through ERK kinases, histamine might induce an intermediate repressor of NF-κB, not excluding CREM repressor isoforms, to facilitate an anti-inflammatory action in SOD1-G93A microglia. Interestingly, the anti-inflammatory response of SOD1-G93A microglia to histamine differed from that observed in non-transgenic microglia, where histamine instead elicited a pro-inflammatory effect *in vitro*, in line with what previously established (10, 11, 25, 41). This would therefore indicate that the response of microglia to histamine is perhaps subjected to the inflammatory context of the environment (10, 13). Our hypothesis of a protective role of histamine in SOD1-G93A microglia is finally coherent with the neuroprotection exerted by histamine on dopaminergic neurons of the *substantia nigra* exposed to the neuroinflammatory LPS (13). Accordingly, when the endogenous synthesis of histamine is genetically prevented in HDC knockout mice, microglia undergo a boosted inflammatory reaction (5), thus reinforcing our hypothesis. As a crucial goal in ALS research is to find strategies to modulate the neuroinflammatory response, in light of our results, we might prospect histamine as an anti-inflammatory trigger at least in ALS microglia.

Because histamine receptor expression is known to change during development (42), and the microglial transcriptome is different in neonatal compared to adult mice (43, 44), and during ALS disease progression (45), it should be of interest to investigate in further studies if the effects of histamine obtained in neonatal SOD1-G93A microglia might be confirmed in adult microglia and moreover be dependent on the specific expression pattern of histaminergic receptors and enzymes. Preliminary studies in spinal microglia cultures from symptomatic ALS mice have shown that histamine indeed downregulated NOX2 expression as in neonatal cultures (Apolloni, personal communication).

With the aim of corroborating the potential modulation and role of the histaminergic system also *in vivo*, in this work, we investigated the expression of the histaminergic system in the cortex and spinal cord of both SOD1-G93A mice and sporadic ALS patients. By analyzing histaminergic expression data as a function of disease progression in SOD1-G93A mice, a significant downregulation of H1R and HDC expression emerged in the lumbar spinal cord at presymptomatic phase. Since H1R activation exerts neuroprotection against excitotoxicity by facilitating glutamate clearance through upregulation of the glutamate transporter 1 (46), we think that the decrease of H1R might facilitate the glutamate-evoked excitotoxicity occurring in ALS mouse spinal cord (47). Moreover, recent studies about HDC knockout mice demonstrate that histamine deficiency reduces the ramified/surveilling morphology of microglia and the production of anti-inflammatory factors. Impaired histaminergic signaling in HDC knockout mice also causes a higher propensity to inflammation, setting the conditions for pathogenic dysregulation of neuroimmune reactions (5). Therefore, the decreased HDC that we demonstrated in SOD1-G93A lumbar spinal cord might likely participate to the exacerbated inflammatory response and enhanced recruitment of immune cells that occur in SOD1-G93A mice (48). In other words, a decreased H1R and HDC expression in spinal cord might thus contribute to generate ALS vulnerability. As the disease moves to the symptomatic phase, always in the lumbar spinal cord we detected an increase of the histamine degrading enzymes HNMT and DAO, which could contribute to a further shortage and impairment of the histaminergic system. In parallel to the persistent downregulation of H1R, we observed the upregulation of the H4R. Because H4R controls the infiltration of regulatory T cells (49) for instance during the acute phase of experimental allergic encephalomyelitis (50), its increase in SOD1-G93A symptomatic mice might indeed be consistent with the suppressive action of regulatory T cells that occurs also in ALS mice (51), and with the inhibition of pro-inflammatory microglia in the course of the disease. At the end stage, while HDC reversed its expression levels, perhaps as a feedback mechanism to balance the endogenous histamine content, the expression of H2R was found decreased. Because H2R activation reduces excitotoxicity and oxidative stress (6), we interpret H2R downregulation as a possible further cause for neuronal damage evoked by increased excitotoxicity and redox dysregulation in ALS.

In cortex respect to spinal cord, we obtained very similar results about HNMT and H2R expression, results only in part confirmatory about HDC and H4R, and opposite data for H1R levels. While at the moment we are unaware of the pathological significance and impact of this discrepancy, we do not exclude a differential contribution of CNS resident and infiltrated cell phenotypes in cortex versus spinal cord. Further studies will hopefully help to decode the tissue and time dependency of the histaminergic dysregulation in SOD1-G93A mice. The only receptor that was not found affected during disease progression in either cortex or spinal cord was the H3R auto receptor that, however, was induced in the hypothalamus at end stage. Because H3R regulates the histamine release from histaminergic neurons (1), its upregulation in SOD1-G93A mice at end stage might confirm an impaired release of histamine and consequent histaminergic deficiency occurring at the hypothalamic level.

Although the modulation of expression of receptors and enzymes is mainly descriptive at this stage, we postulate that upregulation of histamine signaling through receptor modulation might play a role in ALS disease prevention. Moreover, we suggest a potential correlation between histaminergic transmission and inflammatory events occurring in SOD1-G93A mice. In parallel with an increased neuroinflammatory state clearly found in spinal cord of SOD1-G93A mice at symptomatic phase, we observed an increase of HNMT and DAO likely signifying a concomitant impaired histaminergic signaling. Accordingly, when we enhanced the histaminergic transmission through an acute treatment in SOD1-G93A mice with the histamine precursor histidine, our data demonstrated a partial decrease in microgliosis and pro-inflammatory NOX2 and NF-κB expression in ALS mice spinal cord.

Despite cross-species heterogeneity, we observed an extensive modulation of histaminergic gene expression also in sALS patients. In autoptic human cortex, the overall mRNA alteration of histamine metabolizing enzymes was accompanied by an increase of H1R and a decrease of H3R. Because H1R is involved in neuroprotection against glutamate excitotoxicity and oxidative stress (6), we could interpret this increase as a failed attempt to resolve excitotoxicity and oxidative damage occurring in ALS. A similar increase was reported also after ischemia where H1R expression seems to mitigate tissue damage (52). Because activation of H3R auto receptors inhibits histamine synthesis and release (1), its downregulation in the cortex of sALS patients might concur to boost the histaminergic signaling and its neuroprotective effects (53). Conversely, because histamine reduces excitotoxicity also *via* H2R activation (20, 54), the decreased H2R mRNA that we observed in sALS, similar to what referred in mice, might be causative of neuronal damage sustained by increased excitotoxicity (7, 9).

Although these results overall suggest that the histaminergic system is highly dysregulated in ALS, its etiopathogenetic role in still unknown. It will be of paramount importance for instance to pharmacologically manipulate the histaminergic system to dissect its function in ALS pathogenesis.

In conclusion, by reporting the anti-inflammatory action of histamine in ALS microglia and the histamine-related gene modulation in the CNS of ALS patients, we propose the histaminergic signaling as a novel mechanism for understanding ALS, and the histamine pathway as a potential target for therapy.

## Ethics Statement

Animal procedures were performed according to European Guidelines for use of animals in research (2010/63/EU) and requirements of Italian laws (D.L. 26/2014), and approved by the Animal Welfare Office, Department of Public Health and Veterinary, Nutrition and Food Safety, General Management of Animal Care and Veterinary Drugs of the Italian Ministry of Health (protocol number 319/2015PR). All efforts were made to minimize animal suffering and the number of animals necessary to produce reliable results.

## Author Contributions

Study concept and design: SAp and CV. Data acquisition and analysis: SAp, PF, SAm, GN, VV, GM, RI, SC, and CV. Contribution to the collection and selection of tissue samples and/or clinical data: EA. Manuscript and figures drafting: SAp, PF, SAm, GM, RI, SC, and CV. Edited and approved the final version of the manuscript: all authors.

## Conflict of Interest Statement

The authors declare that the research was conducted in the absence of any commercial or financial relationships that could be construed as a potential conflict of interest.

## Abbreviations

ALS, amyotrophic lateral sclerosis; HDC, histidine decarboxylase; HNMT, histamine *N*-methyltransferase; DAO, diamine oxidase; NOX2, NADPH oxidase 2; sALS, sporadic ALS; WT, wild-type.
