# Supplementary figures and images for: Histamine Regulates the Inflammatory Profile of SOD1-G93A Microglia and the Histaminergic System Is Dysregulated in Amyotrophic Lateral Sclerosis
Source: Front Immunol. 2017 Nov 30;8:1689. doi: 10.3389/fimmu.2017.01689 (PMC5714870; doi:10.3389/fimmu.2017.01689)

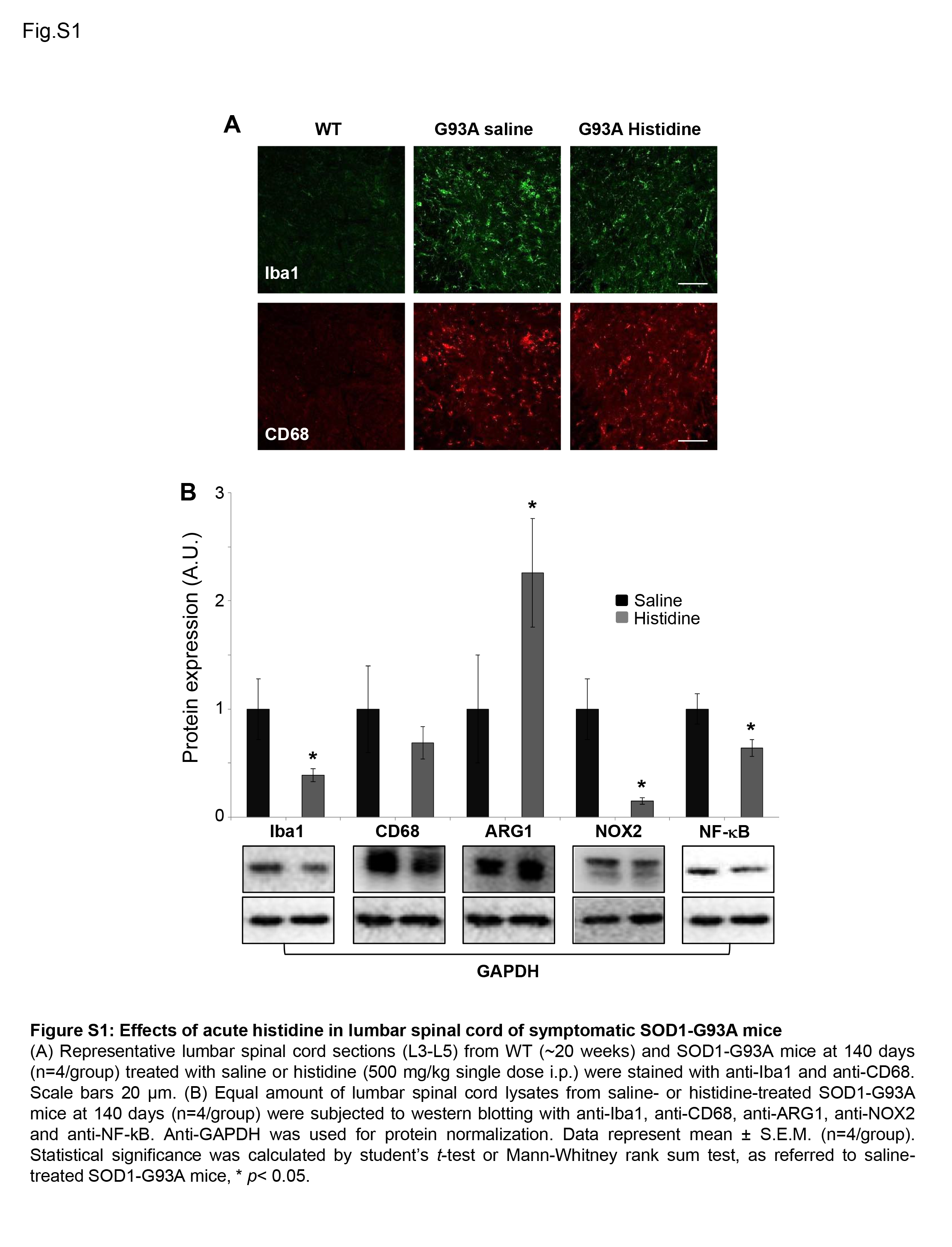

Supplement: Supplementary file 1 [file Image_1.tif]

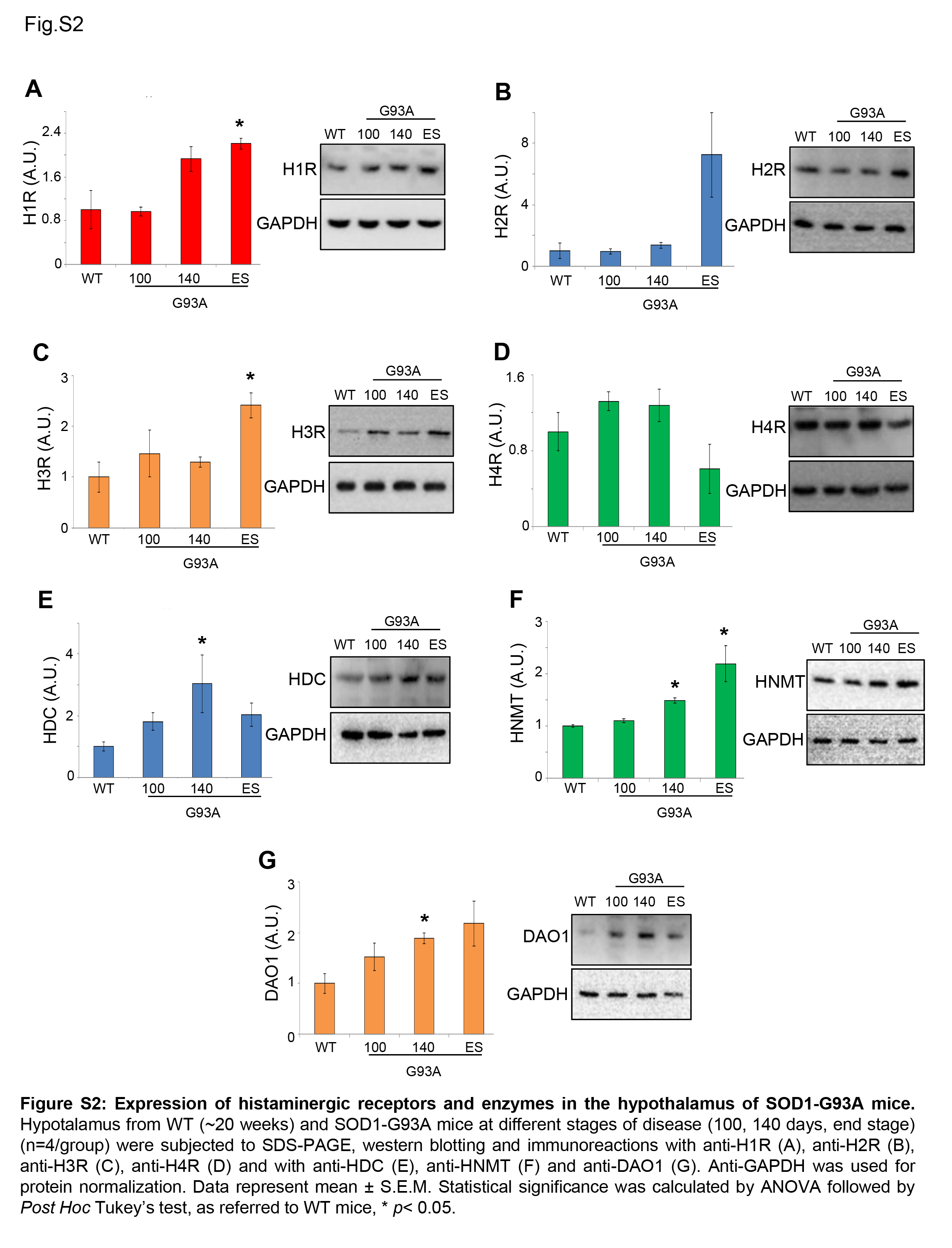

Supplement: Supplementary file 2 [file Image_2.tif]
